# Long-Chain Polyunsaturated Fatty Acids (LCPUFAs) and the Developing Immune System: A Narrative Review

**DOI:** 10.3390/nu13010247

**Published:** 2021-01-16

**Authors:** Elizabeth A. Miles, Caroline E. Childs, Philip C. Calder

**Affiliations:** 1 Faculty of Medicine, School of Human Development and Health, University of Southampton, Southampton SO16 6YD, UK; e.a.miles@soton.ac.uk (E.A.M.); c.e.childs@soton.ac.uk (C.E.C.); 2 Institute for Life Sciences, University of Southampton, Southampton SO17 1BJ, UK; 3 NIHR Southampton Biomedical Research Centre, University Hospital Southampton NHS Foundation Trust and University of Southampton, Southampton SO16 6YD, UK

**Keywords:** immunity, infection, allergy, asthma, inflammation, polyunsaturated fatty acid, fish oil, pregnancy, lactation, infant

## Abstract

The immune system is complex: it involves many cell types and numerous chemical mediators. An immature immune response increases susceptibility to infection, whilst imbalances amongst immune components leading to loss of tolerance can result in immune-mediated diseases including food allergies. Babies are born with an immature immune response. The immune system develops in early life and breast feeding promotes immune maturation and protects against infections and may protect against allergies. The long-chain polyunsaturated fatty acids (LCPUFAs) arachidonic acid (AA) and docosahexaenoic acid (DHA) are considered to be important components of breast milk. AA, eicosapentaenoic acid (EPA) and DHA are also present in the membranes of cells of the immune system and act through multiple interacting mechanisms to influence immune function. The effects of AA and of mediators derived from AA are often different from the effects of the n-3 LCPUFAs (i.e., EPA and DHA) and of mediators derived from them. Studies of supplemental n-3 LCPUFAs in pregnant women show some effects on cord blood immune cells and their responses. These studies also demonstrate reduced sensitisation of infants to egg, reduced risk and severity of atopic dermatitis in the first year of life, and reduced persistent wheeze and asthma at ages 3 to 5 years, especially in children of mothers with low habitual intake of n-3 LCPUFAs. Immune markers in preterm and term infants fed formula with AA and DHA were similar to those in infants fed human milk, whereas those in infants fed formula without LCPUFAs were not. Infants who received formula plus LCPUFAs (both AA and DHA) showed a reduced risk of allergic disease and respiratory illness than infants who received standard formula. Studies in which infants received n-3 LCPUFAs report immune differences from controls that suggest better immune maturation and they show lower risk of allergic disease and respiratory illness over the first years of life. Taken together, these findings suggest that LCPUFAs play a role in immune development that is of clinical significance, particularly with regard to allergic sensitisation and allergic manifestations including wheeze and asthma.

## 1. Introduction

The role of the immune system is to provide protection against pathogenic organisms including bacteria, viruses, fungi and parasites. In order to deal with the potential wide array of threatening organisms, the human immune system has evolved to include many different cell types, many communicating molecules and multiple functional responses. The immune system has four general actions. Firstly, it acts as a barrier keeping microbes from entering the body. Examples of barriers include the skin; the mucosal lining of the gastrointestinal (GI), respiratory and genitourinary tracts; the acid pH of the stomach which kills many bacteria; and anti-microbial proteins in secretions such as tears and saliva. Secondly, the immune system acts to recognise microbes and to identify whether they are harmful or not. Recognition can be of general structural features of microbes or of specific and unique microbial antigens. Thirdly, the immune system acts to eliminate those microbes identified as being harmful; this involves the destructive actions of various types of immune cell. Fourthly and finally, the immune response generates immunological memory. This involves long-term maintenance of memory T lymphocytes (T cells) and B lymphocytes (B cells) so that if there is re-exposure to the harmful microbe, the immune response is more rapid and stronger than it was for the original response. The generation of immunological memory is the basis of vaccination. These complex and sophisticated actions can be achieved because the human immune system is comprised of many cell types, each with their own individual functional capabilities. These different cell types interact with one another as part of the immune response to assure effective protection of the host from pathogens. The immune system may be classified in different ways, most commonly into innate (or natural) and acquired (or adaptive) immunity (Table 1). 

Innate immunity includes the barrier functions, the inflammatory response, and the cells involved in recognition of general structural features of microbes and their subsequent destruction. Acquired immunity includes antigen recognition and antigen-specific effector functions such as the proliferation of T cells, the killing of virally-infected cells by cytotoxic T cells, and the production of antibodies by B cells. Acquired immunity can be further sub-classified into cell-mediated immunity involving T cells and humoral immunity involving B cells and antibody production. Innate and acquired immunity are linked. Phagocytic cells such as macrophages and dendritic cells, which are part of innate immunity, act as antigen-presenting cells, whereby they process and present antigens derived from engulfed microbes to antigen-specific T cells so eliciting acquired immunity. Conversely, cytokines produced by activated T cells regulate the activity of innate immune cells. Thus, there is bidirectional communication between innate and acquired immunity and this can involve both cell-to-cell contact and production of, and responses to, chemical mediators. 

It is obvious that effective defence against pathogenic organisms requires a well-functioning immune system. Consequently, individuals with weakened immune systems are at increased risk of becoming infected and of infections being more serious. The immune system also plays a role in assuring immunologic tolerance of non-threating exposures including harmless microbes (e.g., commensal bacteria in the GI tract) and food components. A breakdown in tolerance to such normally harmless exposures is linked to various diseases including inflammatory bowel diseases (suggested to involve loss of tolerance to commensal gut microbes [1]) and food allergies (loss of tolerance to food components [2]). These diseases involve an adverse inflammatory response that includes actions of both innate and acquired immunity. The immune system develops early in life (see Section 2) and optimal development would generate an immune system that provides robust host defence against harmful microbes and assures tolerance to harmless microbes, to foods and to other environmental exposures. Poor or skewed immune development could generate an immune system that does not provide adequate host defence and/or does not provide adequate tolerance; both these situations could result in disease. Cells of the immune system have a high content of long-chain polyunsaturated fatty acids (LCPUFAs) in their membranes (see Section 4). These LCPUFAs have different biological roles in membranes that influence immune cell function and inflammatory processes, and modification of the LCPUFA content of immune cell membranes can impact on immune function (see Section 4). Therefore, acquisition of different amounts of LCPUFAs by immune cells as they are developing could influence immune maturation and function and this could have a lasting effect on immune competence and risk of diseases involving immune dysfunction. This narrative review will provide an overview of immune development in humans and the importance of factors in breast milk, the role of LCPUFAs and their bioactive metabolites in immunity and inflammation, and human studies investigating the influence of early life exposure to LCPUFAs on immunity, allergic disease and infections.

## 2. Immune Development in Humans 

All cells of the human immune system develop in the bone marrow. Most immune cells also mature in the bone marrow, but T cells mature in the thymus. Immune cells circulate in the bloodstream and in the lymph and are found organised in discrete secondary lymphoid organs such as the spleen and lymph nodes where they interact with one another. Mucosal barriers (e.g., the GI tract, the respiratory tract) also contain organised aggregations of immune cells. It is estimated that in humans 70% of immune cells are associated with the GI tract, mainly in discrete structures such as lamina propria and Peyer’s patches [3]. The reason for such a large congregation of immune cells at mucosal barriers is that these are sites of high exposure to pathogens. 

**Figure 1:**
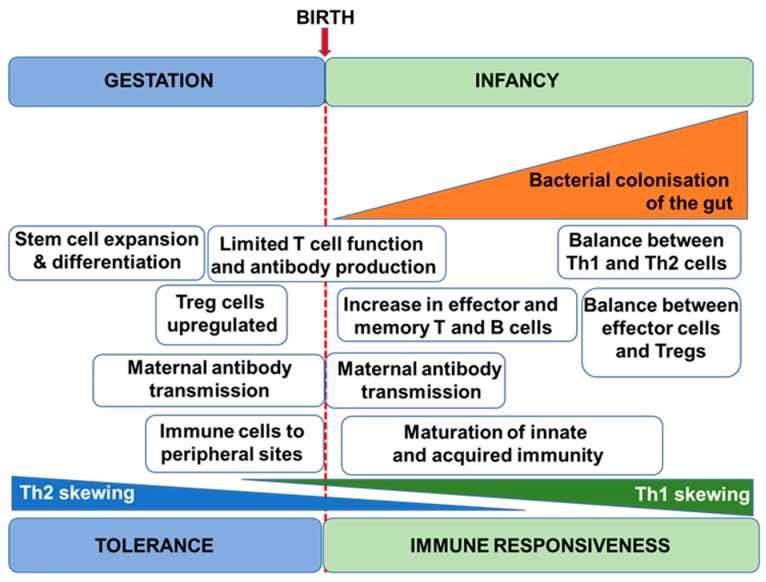
Schematic depiction of the early development of the human immune system. Treg, regulatory T cell.

**Figure 2:**
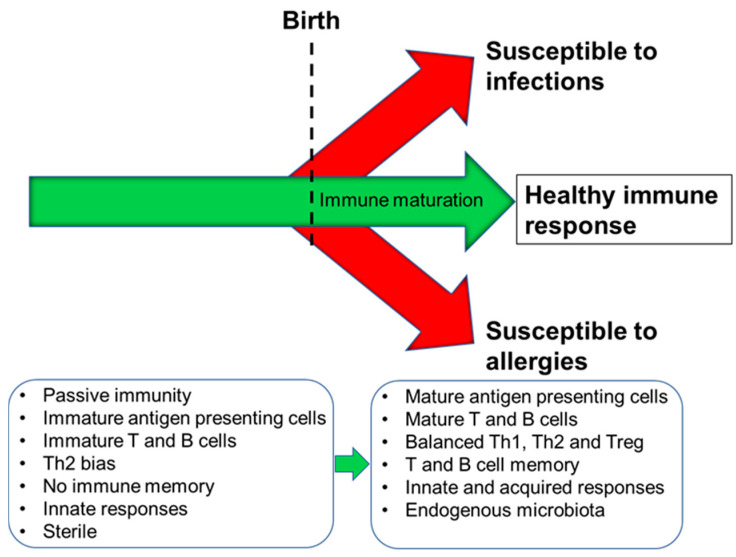
Schematic depiction of immune maturation and the possible outcomes. Th1, T helper 1; Th2, T helper 2; Treg, regulatory T cell.

The human immune system begins to develop before birth with generation of a variety of immune cells and the population of the spleen and lymph nodes (Figure 1). Nevertheless, foetal immune cells are immature with limited functionality [4]. Importantly therefore, the pregnant mother provides passive immunity to the foetus through placental transfer of antibodies [5]. Pregnancy is associated with immune changes in the mother with a dampening of T-helper 1 (Th1)-type responses in favour of T-helper 2 (Th2)-type responses [6,7]; this is to assure maternal tolerance of the foetus. This Th2-skewing is also seen in the developing foetal immune system. Since Th1-type responses are involved in anti-bacterial and anti-viral immunity, pregnant women are at increased risk of bacterial and viral infections, as are newborn infants. After giving birth, the maternal immune system must reverse the pregnancy-associated Th2 skewing, while the newborn infant’s immune system must develop its Th1 competence. The newborn infant has an immature immune system, and maternal transfer of antibodies and other protective molecules in breast milk is important in reducing risk of infection [8]. The newborn’s immune system develops over the course of months to a few years with acquisition of T cell and B cell function and antibody production and the establishment of balances between Th1 and Th2 cells and between these effector T cells and regulatory T cells [9]. Breast milk-derived factors play important roles in this early life immune development (see Section 3) but exposure to antigens (e.g., from microbes and from foods) is also important, as is the acquisition of the infant gut microbiota. In turn, this is affected by the birthing process, by contact with maternal skin, by breast milk factors, and by environmental exposures [10,11,12]. Ultimately, if an appropriate combination of immune maturation factors has been present, the infant develops an effective and balanced immune system that affords both protection against pathogens and tolerance of harmless environmental exposures (Figure 2). Conversely, impaired immune development leading to poor cellular responses or on-going immune imbalances (e.g., between the Th1 and Th2 systems) can result in enhanced infant susceptibility to infections or in development of immune-mediated diseases such as food allergies (Figure 2). 

## 3. Importance of Breast Milk Factors to Immune Development

Human breast milk contains immune cells (neutrophils, macrophages, and T and B cells) and numerous immune-active molecules and immune maturation factors [8,13,14]. These include immunoglobulins (Igs) such as IgG, IgM and secretory IgA; anti-bacterial proteins like lactoferrin, lysozyme and complement C3; anti-viral mucins; many cytokines, chemokines and growth factors; and nucleotides, gangliosides and oligosaccharides. Some of these factors are also involved in promoting a healthy gut microbiota and the co-development of a healthy gut microbiota and a well-functioning immune system seems likely to be promoted through the dual action of breast milk derived factors [11,12,15,16]. The concentrations of immune factors in human breast milk change with duration of the period of lactation; for example, the concentrations of IgG, IgM and IgA are much higher in the first few days of lactation than later [14]. Similarly the total number of immune cells in human breast milk is highest in colostrum and early milk [14]. Human breast milk also contains LCPUFAs which have roles in the immune system. Breast feeding protects against childhood infections [17] and may protect against childhood allergies and asthma [18], and part of this protection seems likely to be due to optimised immune development in breast-fed infants. 

## 4. Long-Chain Polyunsaturated Fatty Acids, Lipid Mediators, Immunity and Inflammation

**Figure 3:**
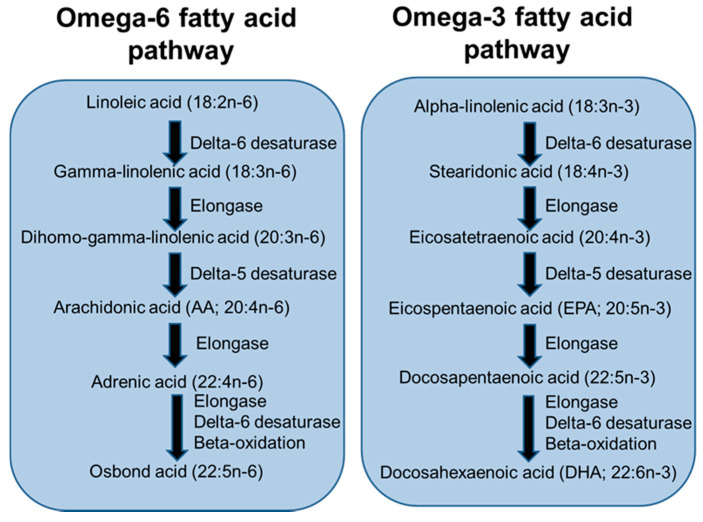
Pathway of long-chain polyunsaturated fatty acid (LCPUFA) biosynthesis from essential fatty acid precursors.

LCPUFAs are considered to be the 20- and 22-carbon chain PUFAs. These are members of the omega-6 (n-6) and omega-3 (n-3) fatty acid families. The main n-6 LCPUFA is arachidonic acid (AA) while the main n-3 LCPUFAs are eicosapentaenoic acid (EPA) and docosahexaenoic acid (DHA). These LCPUFAs are synthesised from precursor essential fatty acids (linoleic acid and α-linolenic acid, respectively) by the pathway depicted in Figure 3. The essential fatty acids are consumed in the diet from many seeds, nuts, vegetable oils and vegetable oil-based spreads. AA is consumed from meat and eggs, while EPA and DHA are consumed from seafood, especially fatty (or “oily”) fish, and from supplements (“fish oils”). AA and DHA are the main LCPUFAs in human breast milk [19] and have been identified to have important roles in development of the infant visual and cognitive systems [20].

**Figure 4:**
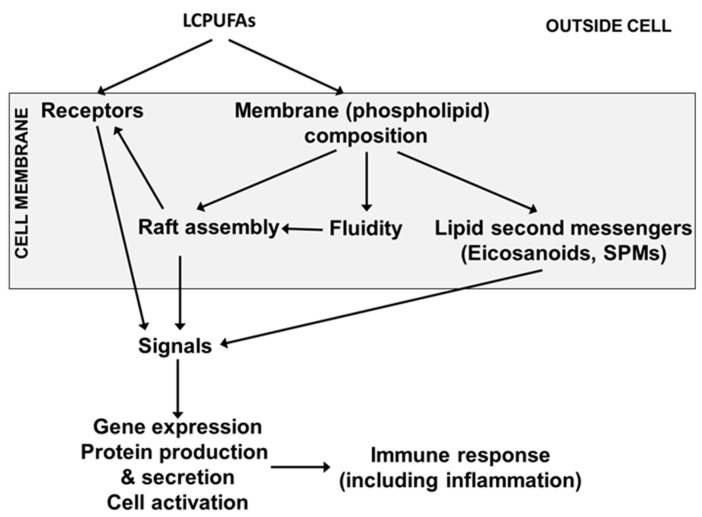
Mechanisms by which LCPUFAs can affect immune cell function SPM, specialised pro-resolving mediator.

Immune cell membranes contain AA, EPA and DHA. AA is the most abundant of these LCPUFAs, while DHA is usually the most abundant n-3 LCPUFA. In adults, AA typically comprises 15 to 20% of total fatty acids in blood mononuclear cells (a mixture of lymphocytes and monocytes) while EPA and DHA typically comprise 0.5 to 1% and 2 to 3%, respectively [21,22]. In umbilical cord blood mononuclear cells from 40 births in Southampton, UK, the mean percentages of AA, EPA and DHA were 17.5, 0.3 and 3.8%, respectively (authors unpublished data). Because of their highly unsaturated nature, LCPUFAs influence the physical nature of cell membranes (sometimes called membrane fluidity) and the function of membrane proteins, including their ability to move within membranes to form signalling platforms termed “lipid rafts” [23] (Figure 4). Hence, LCPUFAs modulate intracellular signalling within immune cells ultimately affecting transcription factor activation and gene expression [23] (Figure 4).

**Figure 5:**
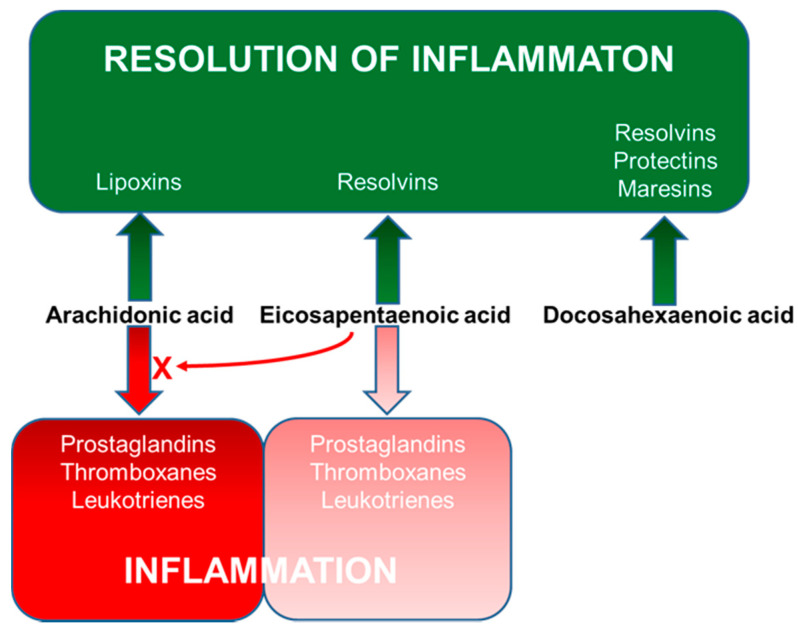
Overview of bioactive lipid mediator synthesis from LCPUFAs.

As a result of these effects, LCPUFAs have been reported to regulate the function of many immune cell types including neutrophils, monocytes, macrophages, dendritic cells, T cells and B cells [24]. Perhaps the best described function of LCPUFAs with regard to immune function, including the inflammatory component, is their role as substrates for the generation of bioactive lipid mediators [25,26] (Figure 5).

AA, EPA and DHA are located within various phospholipids in cell membranes, usually at the *sn*-2 position. They can be released upon cell stimulation, typically as a result of the activity of phospholipase A_2_. Once released, LCPUFAs can enter the cyclooxygenase (COX), lipoxygenase (LOX) or cytochrome P450 pathways [25,26]. The COX pathway gives rise to 2-series prostaglandins (PGs) and thromboxanes from AA, and the 5-LOX pathway gives rise to 4-series leukotrienes (LTs). Several of these AA-derived mediators influence immunity. For example, PGE_2_, which is produced by many immune cell types, has effects on acquired immunity [27,28,29,30], inhibiting dendritic cell interactions with Th1 cells, cytotoxic T cells and natural killer cells. With regard to effects on T cells, PGE_2_ is generally regarded as immunosuppressive, since it decreases T cell proliferation, the production of important cytokines such as interleukin (IL)-2 and interferon (IFN)-γ, the differentiation of naïve T cells to Th1 cells and cytotoxic T cells, and the killing functions of cytotoxic T cells [29,30]. PGE_2_ also inhibits natural killer cell activity [29,30]. Many of these inhibitory effects of PGE_2_ may involve the induction of regulatory T cells [29] which inhibit Th1 and cytotoxic T cell activity. Since dendritic cells, Th1 cells, cytotoxic T cells and natural killer cells are central to host defence against pathogens, especially bacteria and viruses, these effects of PGE_2_ may be considered to be deleterious. The Th1 cell response is balanced with the Th2 type response that involves IL-4, -5 and -13 and is part of defence against extracellular parasites like helminthic worms. PGE_2_ promotes the Th2 type immune response [28,29,30]. The balance of Th1 and Th2 cells affects B cell function and antibody production. Both through its effects on Th2 cells and through direct effects on B cells, PGE_2_ promotes Ig class switching to favour production of IgE [31] which is involved in allergic responses. PGE_2_ induces differentiation of pro-inflammatory T-helper 17 cells [32]. PGE_2_ is also directly involved in inflammation where it has pro-inflammatory roles such as inducing fever and pain and enhancing vascular permeability, which allows neutrophils and macrophages to enter sites of infection or inflammation [27,28]. PGE_2_ also potentiates the pain response induced by other mediators such as bradykinin and histamine [27]. Because of these actions, many anti-inflammatory pharmaceuticals have been developed to target the COX pathway with the aim of decreasing PGE_2_ production [26]. 

Although many of the actions of PGE_2_ are clearly pro-inflammatory and pro-allergic, it suppresses 5-LOX, inhibiting synthesis of LTs (which are generally pro-inflammatory: see below) and induces 15-LOX to promote the synthesis of pro-resolution lipoxins [33,34]. In this regard PGE_2_ acts to trigger resolution of inflammation. This may explain paradoxical observations related to PGE_2_ such as the phenomenon of aspirin-sensitive (or aspirin-exacerbated) respiratory disease, where the condition is made worse by blocking PGE_2_ production [35]. Since PGE_2_ normally prevents excess production of LTs, inhibition of PGE_2_ production by aspirin and other non-steroidal anti-inflammatory drugs permits over-production of the LTs involved in respiratory inflammation [36]. A recent study demonstrated that patients with anaphylaxis had low serum concentrations of PGE_2_ compared to healthy subjects and that PGE_2_ concentrations correlated inversely with anaphylaxis severity [37]. In parallel studies in mice, stabilisation of PGE_2_ concentrations protected against anaphylaxis [37]. Given these observations, it is perhaps more appropriate to consider PGE_2_ as both a mediator and a regulator of inflammation and immunity than simply as immunosuppressive, pro-inflammatory and pro-allergic. 

PGD_2_ is a pro-allergic and pro-inflammatory mediator released from mast cells [28,38]. It has a significant pro-inflammatory effect that is prominent in allergic airways disease (asthma), where it up-regulates some of the distinct characteristics including eosinophilia, airway hyperreactivity, production of mucus, and production of Th2-type cytokines [39]. PGD_2_ also has pro-inflammatory effects in the skin where it promotes erythema, oedema, induration, and leukocyte infiltration [40]. As a vasodilator, PGD_2_ contributes to inflammation by increasing local blood flow. PGD_2_ also has effects within acquired immunity but these are not well described, although, like PGE_2_, it has been reported to suppress the Th1-mediated response [30].

The metabolites of AA produced by LOX enzymes are also important in inflammation and immunity [28,41,42,43,44,45]. LTB_4_, produced by the 5-LOX pathway, is generated by many different cell types, particularly neutrophils and macrophages and also by epithelial cells. LTB_4_ is a bronchoconstrictor, increases vascular permeability and acts as a strong chemoattractant, acting to recruit leukocytes (especially neutrophils and macrophages) to sites of inflammatory or immune activity [28,41,42,46]. LTB_4_ also enhances the adhesion of leukocytes to the endothelium and induces various target cells, particularly neutrophils, to produce of reactive oxygen species and proteolytic enzymes. LTB_4_ also induces production of pro-inflammatory cytokines by neutrophils and macrophages. Thus, the effects of LTB_4_ are pro-inflammatory. LTB_4_ also influences the function of T cells (increased proliferation and IL-2 and IFN-γ production, suggesting enhanced Th1 cell function), B cells (differentiation and IgE production) and natural killer cells (enhanced cytotoxicity) [46]. Hence, LTB_4_ seems to promote some components of immune defence.

The 5-LOX pathway also generates cysteinyl-LTs (LTC_4_, D_4_ and E_4_) from AA primarily by basophils, eosinophils, mast cells and macrophages. This is in response to a range of stimuli, including allergens. Thus, the cysteinyl-LTs are strongly linked to the allergic response. Cysteinyl-LTs enhance vascular permeability, promote eosinophil recruitment to the airways and are potent inducers of smooth muscle contraction, including in the airways (bronchoconstriction) where they have a role in asthma [28,41,42,44,47]. They also induce mucus production by goblet cells and oedema and activate various pro-inflammatory responses of mast cells, macrophages and neutrophils, including production of reactive oxygen species, cytokines and proteases. Cysteinyl-LTs promote the Th2 response, with LTE_4_ being the most active.

N-3 LCPUFAs (EPA and DHA) decrease the production and concentrations of eicosanoids derived from AA [22,48,49,50,51]. This occurs partly as a result of reduced substrate (AA) availability because the n-3 LCPUFAs partially replace AA in cell membranes [21,22]. EPA is also metabolised in the COX and LOX pathways [26]. Via the COX pathway, EPA is used to produce 3-series PGs and thromboxanes and via the 5-LOX pathway it produces 5-series LTs [26,52] (Figure 5). In contrast to the eicosanoids formed from AA, those formed from EPA frequently have weak actions. For example, whereas PGE_2_ was a potent inhibitor of T cell proliferation in vitro, PGE_3_ had little effect [53]. Similarly, LTB_4_ is 10- to 100-fold more potent as a leukocyte chemoattractant than LTB_5_ is [54,55].

One of the most significant developments in the understanding of the actions of n-3 LCPUFAs has been the discovery that new families of lipid mediators are produced from both EPA and DHA by the COX and LOX pathways. These mediators are collectively termed “specialised pro-resolving mediators” (SPMs) since they resolve (“turn off”) on-going inflammation [56,57,58]. Several SPMs also promote aspects of innate immunity including phagocytosis of bacteria and of cellular debris [59]. SPMs include resolvins, protectins (also known as neuroprotectins) and maresins. EPA and DHA give rise to the E- and D-series resolvins, respectively, while both the protectins and maresins are produced from DHA [56,57,58,59] (Figure 5). Many immune cell types produce SPMs [56,57,58,59]. SPMs have been measured in human plasma including from pregnant women [60], umbilical cord [60,61,62], infants [61] and children [61,62]. Human breast milk has been reported to contain SPMs [63,64,65]. Increased intake of EPA and DHA leads to increased amounts of EPA and DHA in immune cells [21,22,66,67], to increased production of SPMs and to higher concentrations of a number of SPMs in the bloodstream (reviewed recently in [68]), although this has not yet been clearly demonstrated in pregnancy or infancy. 

When the biological actions of the different lipid mediators formed from the n-6 LCPUFA AA and from the n-3 LCPUFAs EPA and DHA are considered it would seem that a balanced supply of precursors would be important in order to achieve “optimal” immune cell membrane contents of the various LCPUFAs (although what exactly constitutes “optimal” is currently unclear) and that this would contribute to an immune system that helped in protection against pathogens whilst avoiding the adverse effects of exaggerated inflammation.

## 5. LCPUFAs, Immune Development, Allergic Disease and Infection 

### 5.1. Trials of n-3 LCPUFAS in Pregnant and Lactating Women

Several studies of maternal n-3 LCPUFA supplementation during pregnancy have investigated early biomarkers of immunity and/or allergic and respiratory outcomes in the offspring [69,70,71,72,73,74,75,76,77,78,79,80,81,82,83,84,85,86,87,88]; these studies are summarised in Table 2. Studies started the n-3 LCPUFA intervention as early as 12 weeks of pregnancy [80] or as late as 30 weeks of pregnancy [81,82], although most started between weeks 18 and 25 (Table 2). Most studies continued n-3 LCPUFA supplementation until the women gave birth (Table 2), although two studies extended the supplementation period to 15 or 16 weeks of lactation [74,75,80]. Most studies used combinations of EPA + DHA (Table 2), although two studies used DHA alone [80,87,88]. Studies using the combination of EPA + DHA used supplements where EPA predominated [74,75,81,82,86] or where DHA predominated [69,70,71,72,73,83,84,85]. One study compared an EPA-rich with a DHA-rich formulation which both contained EPA and DHA [76,77,78,79]; however, the doses of EPA and DHA were not perfectly matched between the two formulations. All studies, except one [69], which incorporated the n-3 LCPUFAs into a milk-based supplement, used capsules. Across all the studies the total dose of EPA, DHA and EPA + DHA used varied from 0 to 1.6 g/day, 0.27 to 2.07 g/day and 0.4 to 3.09 g/day, respectively (Table 2). All studies listed in Table 2 involved a control group and in all except one [80] the control group consumed some form of placebo, usually a vegetable oil supplement. Other aspects, not shown in Table 2, such as sample size and the precise health characteristics of the women being studied also differed among the studies. Hence, there is considerable heterogeneity in the study characteristics. Furthermore, there is heterogeneity in the immune markers and health outcomes assessed and the time point at which these assessments were made (Table 2). 

Three of the studies reported that increasing maternal intake of n-3 LCPUFAs modifies immune markers in umbilical cord blood [69,70,71,72,73,76,77,78], with effects generally consistent with a dampening of Th2-type responses, a rebalancing of Th1- and Th2-type responses and promotion of immune maturation (Table 2). However, not all immune parameters that have been measured are affected by increased maternal n-3 LCPUFA intake. Heterogeneity in these outcomes may relate to the dose and exact composition of n-3 LCPUFAs used, which vary greatly across the studies, although the explanation is likely more complex than this. For example, some immune parameters may simply be less sensitive to n-3 LCPUFAs than others. One study reported that maternal DHA supplementation (0.4 g/day) from week 12 of pregnancy until 4 months post-partum alters some immune markers in the infants’ blood at age 4 months [80]. The effects of maternal n-3 LCPUFAs observed in some studies may relate to epigenetic differences, as reported for cord blood immune cells in some studies [89] though not in others [90]. One study investigated whether maternal n-3 LCPUFA supplementation (1.5 g EPA + DHA per day incorporated into muesli bars) solely during the first 4 months of lactation affected immune outcomes in the offspring at age 2.5 years [91]. Cultures of whole blood from the children of mothers who received n-3 LCPUFAs during lactation produced higher amounts of IFN-γ and had a higher ratio of IFN-γ to IL-10 after stimulation with bacterial lipopolysaccharide [91]. The authors interpreted this to indicate faster maturation of the immune system in the children whose mothers consumed n-3 LCPUFAs. 

The effects of early exposure to n-3 LCPUFAs on the immune system reported in some studies (Table 2) might influence allergic sensitisation and the risk of allergic (and also infectious) diseases later in life. In support of this notion, less severe atopic dermatitis and lower risk of sensitisation to egg were seen in one year old infants whose mothers had consumed n-3 LCPUFA supplements providing 1.02 g EPA + 2.07 g DHA daily from week 20 of pregnancy [71]. Some other clinical outcomes were numerically lower in the infants whose mothers had received n-3 LCPUFAs, but the differences were not statistically significant, perhaps because sample size was too small. N-3 LCPUFA supplementation (1.6 g EPA + 1.1 g DHA daily) during both pregnancy and lactation resulted in lower PGE_2_ production by stimulated maternal blood collected within one week of birth [92]. This might influence Th2 polarisation in the foetus. In this study, infants whose mothers had received n-3 LCPUFAs had less risk of becoming sensitised to egg, less IgE-associated eczema (atopic dermatitis) and less food allergy during the first year of life [74]. Over the first two years of life there was a lesser risk of developing any IgE-mediated disease or IgE-associated eczema or being sensitised to egg or to any allergen that was tested in the n-3 LCPUFA group [75]. Receiving n-3 LCPUFAs in late pregnancy (1.28 g EPA + 0.92 g DHA daily) was associated with reduced asthma-related diagnoses in the offspring at age 16 years [81]. This suggests a long-term effect of any immunologic changes that occurred in pregnancy and early life. In fact, some of these effects were still apparent at age 24 years [82]. 

Twelve-month-old infants of mothers who consumed a DHA-rich oil (0.1 g EPA + 0.8 g DHA daily) during pregnancy showed less sensitisation to hens’ egg than the control group [83]. There was also a strong trend to less IgE-associated eczema, but there was no difference in “any IgE-mediated disease” [83]. These infants were followed up at age 3 years: there was no effect of the DHA-rich oil on any clinical outcome including asthma [84]. However, at age 6 years sensitisation to one species of house dust mite was lower in the DHA group than in the control group [85]. Significantly lower incidence of persistent wheeze or asthma at ages 3 to 5 years was seen in children whose mothers took n-3 LCPUFAs (1.32 g EPA + 0.88 g DHA daily) from week 24 of pregnancy [86]. There was also lower risk of respiratory tract infections over the first 5 years of life in the offspring of mothers in the n-3 LCPUFA group [86]. The beneficial effect of maternal n-3 LCPUFAs on offspring persistent wheeze or asthma was seen mainly in the subset of children whose mothers had the lowest EPA + DHA status at study entry and was less apparent in the subset of children whose mothers had the highest EPA + DHA status at study entry [86]. This suggests that n-3 LCPUFAs will be of most benefit to those with the lowest status and may be less effective in those who already have a high status. Further to this, the effect of maternal n-3 LCPUFAs on offspring persistent wheeze or asthma was seen mainly in the subset of children whose mothers had a specific polymorphism (G allele at rs1535) in the fatty acid desaturase 2 gene that is associated with low blood levels of EPA and DHA [86]. Thus, the genetic predisposition to low maternal blood level of n-3 LCPUFAs may result in greater benefit being obtained from supplemental EPA + DHA. A large study evaluated the effect of 0.4 g DHA daily in pregnant women from 18 to 22 weeks gestation until giving birth: infants whose mothers were in the DHA group showed reduced cold symptoms at age one month, less illness time to age 3 months and reduced illness at age 6 months [87]. In that study, infants of atopic mothers who received DHA showed lower likelihood of respiratory illness up to age 18 months [88].

In contrast to the favourable effects of n-3 LCPUFAs seen in many of these studies (Table 2), one study found significantly increased atopic dermatitis in three-year-old children whose mothers had received either one of two doses of n-3 LCPUFAs during pregnancy [79]. This study did demonstrate effects of n-3 LCPUFAs on immune markers in both the mothers and in cord blood that would be consistent with a reduced Th2 phenotype and a reduced predisposition to allergic responses [76,77,78]. Therefore, the reasons for the clinical findings are unclear, but highlight that more needs to understood about this area. 

### 5.2. Trials of LCPUFAs in Infants

There are relatively few trials of LCPUFA intervention in infants with immune system follow-up. Field et al. [93] compared human milk with standard formula or standard formula with added AA (0.49% of fatty acids) and DHA (0.35% of fatty acids) in 44 preterm infants. The duration of intervention was 42 days. An age-related increase in T-helper cells and in B cells was seen in the human milk and the formula + LCPUFA groups but not in the standard formula group. Likewise, in the human milk and formula + LCPUFA groups more T cells were antigen mature at day 42 and fewer were antigen naïve. Production of IL-10, a regulatory cytokine, was not different in the human milk and formula + LCPUFA groups but was low in the standard formula group. Conversely, production of the pro-inflammatory cytokine tumour necrosis factor was not different in the human milk and formula + LCPUFA groups but was lower than in the standard formula.

The findings of Field et al. [93] suggest that inclusion of AA and DHA in formula results in an immune profile that is consistent with that seen with human milk, suggesting that some of the immune effects of breast milk are due to its component LCPUFAs. This same group of researchers enrolled 30 term infants into a trial of formula compared with formula + arachidonic acid (AA) (0.34% of fatty acids) and docosahexaenoic acid (DHA) (0.20% of fatty acids) [94]. The infants were aged 2 weeks and the duration of the trial was 4 weeks. There was also a breast-fed comparator group. Compared to formula-fed infants, infants fed formula + LCPUFAs had a blood immune cell distribution and a blood cytokine profile that was not different from those of breast-fed infants. These two studies indicate that LCPUFAs result in an improved immune response in very young infants, although it is not clear if both n-6 and n-3 LCPUFAs need to be present to achieve this effect. These studies did not investigate response to vaccination, infections or immune-mediated illnesses or the persistence of the immune effects reported beyond the end of the intervention period.

Some trials have investigated clinical outcomes in infants who received LCPUFAs in infant formula [95,96,97,98]. Birch et al. [95] followed up term infants who had received standard formula or formula + LCPUFAs (AA 0.64–0.72% of fatty acids and DHA 0.32–0.36% of fatty acids) in two different studies. Infants had received the formulas from age <6 days to 12 months and they were followed up to the age of 3 years. In the group of infants who had received LCPUFAs there was a lower risk of wheezing/asthma, wheezing/asthma plus atopic dermatitis, any allergy and upper respiratory tract infection. In another study [96], term infants received standard formula or formula + LCPUFAs (AA 0.64% of fatty acids and DHA 0.32% of fatty acids) from age ~1 month until one year. Infants who were receiving LCPUFAs were less likely to develop bronchitis or bronchiolitis at 5, 7 and 9 months. A third study used the same comparison [97] and identified that infants who were receiving LCPUFAs were less likely to develop bronchitis or bronchiolitis, croup, nasal congestion, cough or diarrhoea requiring medical attention over the first year of life. Foiles et al. [98] followed-up a subset of infants who had been involved in a trial of different infant formulas for the first 12 months of their life. They were term infants and had received a control formula without LCPUFAs or one of three formulas containing a fixed amount of AA (0.64% of fatty acids) and one of three different amounts of DHA (0.32, 0.64 or 0.96% of fatty acids). Infants receiving LCPUFAs has less allergic illness and less skin allergic illness in the first year compared to infants in the control group and these outcomes remained lower at age 4 years. LCPUFAs also delayed time to first allergic illness and first skin allergic illness and tended to decrease wheeze/asthma. LCPUFAs reduced wheeze/asthma in infants of allergic mothers [98]. 

The effect of n-3 LCPUFAs (0.11 g EPA + 0.28 g DHA daily) given from birth until 6 months of age to infants at high risk of developing allergy on immune outcomes [99] and allergic disease [100] was investigated. Mononuclear cells (a mix of T cells, B cells and monocytes) from infants who had received n-3 LCPUFAs produced less of the Th2 cytokine IL-13 when stimulated ex vivo with house dust mite [99]. These cells also produced more of the Th1 cytokines IFN-γ and tumour necrosis factor when stimulated with phytohaemagglutinin. These observations are consistent with a favourable shift in the Th1 versus Th2 balance after n-3 LCPUFA supplementation that would result in less predisposition to allergy and a better ability to deal with bacteria and viruses. Eczema at age 12 months was predicted by both low plasma DHA and low red blood cell EPA [99]. Nevertheless, clinical outcomes (any allergic disease, total or any specific sensitisation, eczema, food allergy, wheeze) at age 12 months were not different between the n-3 LCPUFA and control groups of infants [100]. However, at age 12 months infants who had been most compliant to the intervention had a lower risk of eczema. Moreover, infants with a higher red blood cell EPA, a higher red blood cell ratio of EPA to AA or a higher plasma DHA at age 6 months were less likely to develop eczema by age 12 months [100]. Furthermore, infants with higher plasma DHA or higher plasma EPA + docosapentaenoic acid + DHA at age 6 months were less likely to develop recurrent wheeze by age 12 months [100]. 

In another study, researchers randomised 64 term infants to cow’s milk or infant formula each without or with added n-3 LCPUFAs (~0.57 g EPA + 0.38 g DHA daily) from age 9 to 12 months [101]. There was no difference between groups in plasma IgE, C-reactive protein, soluble IL-2 receptor or fecal IgA. However, n-3 LCPUFAs resulted in enhanced IFN-γ production in cultures of whole blood that were stimulated with *Lactobacillus paracasei*. The authors concluded that their results indicated better immune maturation in infants given additional n-3 LCPUFAs. 

The long-term effect of n-3 LCPUFA supplementation of infants on allergic disease was studied [102,103,104,105,106]. Infants in the n-3 LCPUFA group received 0.5 g of DHA-rich tuna oil per day (this would provide ~0.25 g DHA per day) added to their formula if they were not being breast fed; if they were being breast fed they did not receive additional n-3 LCPUFAs. The control group received vegetable oil to add to formula if they were not being breast fed. Almost 70% of infants in both groups had some breast feeding; thus, a number of infants in the n-3 LCPUFA group would not have received the n-3 LCPUFA supplement for part of the intervention period and conversely both groups of infants would have been receiving n-3 LCPUFAs from breast milk. Nevertheless, at 18 months of age there was decreased prevalence of wheeze in the n-3 LCPUFA group and higher plasma n-3 PUFA levels were associated with lower bronchodilator use [103,104]. At follow-up at 3 years of age, there was reduced cough, but not wheeze, in the n-3 LCPUFA group but there was no difference between the groups for other outcomes such as eczema, serum IgE concentration or doctor diagnosis of asthma [104]. At later follow up when the children were aged 5 years, there was no difference between groups for any clinical outcome related to lung function [105], allergy [105], or asthma [106]. There are a number of reasons why the early benefits of n-3 LCPUFAs did not persist: these include less than optimal adherence to the intervention (50% and 56% compliance in the intervention and control group, respectively), loss to follow-up, lack of power, the low dose of n-3 LCPUFAs used, and the fact that other, later, exposures may nullify any early effects of n-3 LCPUFAs.

Data from a birth cohort study in Iceland identified that 2.5-year-old children who had received n-3 LCPUFA supplements in infancy were less likely to have been diagnosed with food sensitisation [107]. They also had a lower incidence of challenge-confirmed food allergy, although that failed to reach statistical significance [107]. Those children who began n-3 LCPUFA supplementation in the first 6 months of life were better protected than those who began later (significant for both sensitisation and allergy) [107]. N-3 LCPUFAs also decreased severity of allergy [107].

Taken together, the findings of these studies suggest that immune effects of early life exposure to n-3 LCPUFAs (e.g., promoting an enhanced balance between Th1 and Th2 responses) have benefits in protecting against respiratory disorders (and diarrhoea) beyond the period of n-3 LCPUFA exposure. However, the long-term persistence of such effects is uncertain.

## 6. Summary, Discussion and Conclusions

The immune system is complex, involving many cell types and numerous chemical mediators and immune balances which are vital to health. An immature immune response increases susceptibility to infection, whilst imbalances amongst immune components leading to loss of tolerance can result in immune-mediated diseases including food allergies. Human babies are born with an immature immune response. The immune system develops in early life and breast feeding promotes immune maturation and protects against infections and possibly also against allergies. The LCPUFAs AA and DHA are present in breast milk, with EPA also being present in very low amounts. AA, EPA and DHA are also present in the membranes of cells of the immune system and act through multiple interacting mechanisms to influence immune function. The effects of AA and of mediators derived from AA are often different from the effects of the n-3 LCPUFAs and of mediators derived from them. There has been much interest in the role of LCPUFAs in general, and of n-3 LCPUFAs in particular, in early immune development especially in the context of risk of allergies and asthma. Several studies of n-3 LCPUFAs in pregnant and/or lactating women have been conducted; these have most often used some form of fish oil providing both EPA and DHA. Doses of n-3 LCPUFAs used in these studies have been variable and studies also differ in other characteristics. Effects of n-3 LCPUFAs during pregnancy on cord blood immune cells and their responses have been reported, but it is not known if these effects on the immune system persist. These studies also report that increased intake of n-3 LCPUFAs during pregnancy can reduce sensitisation to common food allergens and decreases the risk and severity of atopic dermatitis in the first year of an infant’s life, with one study indicating a persistence until adolescence and early adulthood. One study has reported that n-3 LCPUFAs in pregnancy decrease the risk of persistent wheeze and asthma in the offspring at ages 3 to 5 years, especially in children of mothers with low status of n-3 LCPUFAs and with a particular polymorphism in the fatty acid desaturase 2 gene. There have also been studies of n-3 LCPUFAs in infants and children, mostly looking at respiratory illness, but the outcomes from these are not clear. Immune markers in preterm and term infants fed formula with AA and DHA were similar to those in infants fed human milk, whereas those in infants fed formula without LCPUFAs were not. Infants who received formula plus LCPUFAs (both AA and DHA) had a lower risk of allergic disease and respiratory illness than those who received standard formula. Studies in which infants received n-3 LCPUFAs report immune differences from controls that suggest better immune maturation and they had lower risk of allergic disease and respiratory illness over the first years of life. Taken together, these observations indicate that LCPUFAs play a role in immune development that is of clinical significance, particularly with regard to allergic sensitisation and allergic manifestations including wheeze and asthma. Studies in pregnancy, lactation and infancy suggest that n-3 LCPUFAs, usually the combination of EPA and DHA, have immune benefits, although the duration of the persistence of the effects is not clear from the current literature because most studies have a limited duration of follow-up. Studies of formulas providing both AA and DHA do not allow for any separate effects of AA compared with n-3 LCPUFAs to be identified, and it is not clear whether AA contributes to the beneficial effects of these formulas on immune-related outcomes. LCPUFAs may contribute to the immune benefits of human breast milk but the extent of this contribution is also unclear. Indeed, whether the effects of LCPUFAs delivered in breast milk, which contains many other immune active components, are the same as LCPUFAs delivered in formula, which lacks many of those components is not known.

It is important to note that the doses of n-3 LCPUFAs used in many of the studies conducted to date are high compared to minimum recommended intakes. For example, the Food and Agricultural Organisation of the United Nations recommends a minimum intake of 0.3 g of EPA + DHA per day, of which at least 0.2 g should be DHA, for pregnant or lactating women [108], while the European Food Safety Authority states an adequate intake of 0.25 g of EPA + DHA per day for adults with an additional 0.1 to 0.2 g DHA per day for pregnant women [109]. The UK recommendation, which is based upon intake of fish, is a minimum intake of 0.45 g EPA + DHA per day [110]. As mentioned previously, the studies in pregnant and/or lactating women described in Table 2 used doses of 0.4 to 3.09 g EPA + DHA per day including 0.27 to 2.07 g DHA per day. It would be difficult to achieve most of these intakes from supplements because a standard “fish oil” supplement provides ~0.3 g EPA + DHA (see Table 2 of [111]). Higher intakes of n-3 LCPUFAs can be achieved by regular consumption of fatty fish, but at least one portion of fatty fish would need to be consumed most days to achieve a daily intake of EPA + DHA of 1.5 g (see Table 1 of [111]) as used in some studies. Thus, the very highest intakes used in the studies described in Table 2 seem unlikely to be achieved from either supplements or from the diet. 

This narrative review has focused on studies that used supplemental n-3 LCPUFAs in pregnant and/or lactating women or in infants. It has not considered studies of fish because fish provides nutrients of relevance to immunity, inflammation and allergic disease risk other than n-3 LCPUFAs including vitamin D, selenium and zinc. 

In conclusion it appears that increased intake of n-3 LCPUFAs in pregnancy may be a useful approach to modulate the infant immune system in order to prevent infant and childhood allergic disease, although in general high intakes of EPA and DHA have been used in studies conducted to date and how these can translate to real-world settings is uncertain. Additional research of the impact of increased provision of n-3 LCPUFAs during pregnancy, lactation, and infancy is needed in order to better identify the immunologic and clinical effects in infants and children and how long these persist.

## Figures and Tables

**Table 1:** Overview of the components of the immune system and their classification into innate and acquired immunity.

Innate (Natural) Immunity		Acquired (Adaptive) Immunity	
Barriers	Cellular Components	Cell-Mediated Immunity	Humoral Immunity
Skin Mucosal surfaces Mucus Antimicrobial proteins in secretions Acid pH of stomach	Granulocytes (Neutrophils, Basophils, Eosinophils, Mast cells) Phagocytes (Neutrophils, Macrophages, Dendritic cells) Inflammatory response Natural killer cells Other innate cells (includes Innate lymphoid cells, Mucosal associated invariant T cells)	T lymphocytes (Helper, Cytotoxic, Regulatory, Others) Cytokines	B lymphocytes Antibodies
		Memory response	

**Table 2:** Summary of randomised controlled trials of n-3 LCPUFAs in pregnant and/or lactating women reporting on immune and related illness outcomes in the offspring.

Reference(s)	Period of Intervention	Intervention (g/day)	Control	Effect of n-3 LCPUFAs on Infant Immune Outcomes	Effect of n-3 LCPUFAs on Infant/Child Clinical Outcomes
[69]	From week 22 of pregnancy until birth	EPA 0.15 plus DHA 0.5 in milk-based supplement	Milk-based supplement without n-3 LCPUFA	Cord blood: Lower CCR4, IL-4 and IL-13 mRNA; Higher TGF-β mRNA; No effect on CRTH2, CXCR3, IL-1 or IFN-γ mRNA	-
[70,71,72,73]	From week 20 of pregnancy until birth	EPA 1.02 plus DHA 2.07 in capsules	Olive oil capsules	Cord blood: Lower IL-13; No effect on numbers of T, helper T, cytotoxic T, B or natural killer cells; Lower LTB_4_ production by neutrophils; Higher percentage of CD34^+^ progenitor cells (but no effect on cytokine or chemokine receptor expression on these cells); Lower IL-10 production by mononuclear cells stimulated with cat allergen or house dust mite allergen (trend with egg allergen); Trends to lower IL-5, IL-13 and IFN-γ production by mononuclear cells stimulated with cat, egg or housedust mite allergen	At one year of age: Less likelihood of severe atopic dermatitis; Trends for less likelihood of SPT positivity to egg and less likelihood of asthma; No effect on likelihood of food allergy, atopic dermatitis, wheeze, any SPT positivity and SPT positivity to allergens other than egg
[74,75]	From week 25 of pregnancy until week 15 of lactation	EPA 1.6 plus DHA 1.1 in capsules	Soybean oil capsules	-	At one year of age: Less likelihood of SPT positivity, SPT positivity to egg, having IgE-associated atopic dermatitis and having food allergy At two years of age: Less likelihood of SPT positivity, SPT positivity to egg or to food, having IgE-mediated food allergy, IgE-associated atopic dermatitis or IgE-associated disease
[76,77,78,79]	From week 12 to 20 until week 34 to 36 of pregnancy	EPA 1.06 plus DHA 0.274 or EPA 0.18 plus DHA 0.9 in capsules	Soybean oil capsules	Cord blood: Lower ratios of Th2 to Th1 chemokines (CCL22:CXCL10 and CCL17:CXCL10) in both n-3 LCPUFA groups; No effect on multiple cytokines; Higher 17-hydroxyDHA in both n-3 LCPUFA groups	At three years of age: Greater likelihood of atopic dermatitis in both n-3 LCPUFA groups
[80]	From week 12 of pregnancy until week 16 of lactation	DHA 0.4 from capsules	None	Blood at 4 months: No effect on IgA, IgM or IgG; No effect on lymphocyte or T helper, memory T helper or natural killer cell numbers; Lower percentage of cytotoxic T cells; Higher percentage of naïve T helper and memory cytotoxic T cells; Lower percentage of IFN-γ and IL-4 producing T helper and cytotoxic T cells in response to phorbol ester and ionomycin stimulation	-
[81,82]	From week 30 of pregnancy until birth	EPA 1.28 plus DHA 0.92 from capsules	Olive oil capsules	-	At 16 years of age: Less likelihood of asthma; allergic asthma; asthma, atopic dermatitis or allergic rhinitis; and allergic asthma, atopic dermatitis or allergic rhinitis At 24 years of age: Less likelihood of allergic asthma, diagnosed asthma, and requiring medication of asthma; Trend to less likelihood of requiring medication of allergic rhinitis; No effect on allergic sensitisation (allergen-specific IgE)
[83,84,85]	From week 21 of pregnancy until birth	EPA 0.1 plus DHA 0.8 from capsules	Blended vegetable oil capsules	-	At one year of age: Trend to less likelihood of IgE-associated allergic disease; Less likelihood of atopic dermatitis and sensitisation to egg; No effect on food allergy or respiratory infections In the first three years of life: Trend to less likelihood of IgE-associated allergic disease and to less likelihood of atopic dermatitis; No effect on food allergy or any sensitisation At three years of age: No effects on any outcome related to allergic disease At six years of age: No effects on any outcome related to allergic disease or on sensitisation to most allergens; Decreased likelihood to be sensitised to one species of house dust mite
[86]	From week 24 of pregnancy until birth	EPA 1.32 plus DHA 0.88 from capsules	Olive oil capsules	-	In the first five years of life: Less likelihood of persistent wheeze or asthma; Less likelihood of respiratory tract infections No effect on atopic dermatitis
[87,88]	From weeks 18 to 22 of pregnancy until birth	DHA 0.4 from capsules	Blended vegetable oil capsules	-	At one month of age: Shorter duration of cough, phlegm or wheezing At three months of age: Shorter duration of nasal congestion and all illnesses At six months of age: Shorter duration of fever, nasal secretion, difficulty breathing, rash and “other” illnesses Over the first 18 months of age: Less likelihood of some respiratory symptoms
[91]	First 4 months of lactation	EPA plus DHA 1.5 incorporated into muesli bars	Olive oil incorporated into muesli bars	Blood at 2.5 years of age: No effect on plasma IgE or IL-10 production by whole blood in response to lipopolysaccharide; Higher IFN-γ production by whole blood in response to lipopolysaccharide and a higher ratio of IFN-γ to IL-10	At 2.5 years of age: No effect on atopic dermatitis, wheezing or food allergy

Abbreviations used: CCL, CC chemokine ligand; CCR, CC chemokine ligand receptor; CRTH, prostaglandin D_2_ receptor; CXC, CXC chemokine ligand; CXCR, CXC chemokine ligand receptor; IFN, interferon; Ig, immunoglobulin; IL, interleukin; LT, leukotriene; SPT, skin prick test; TGF, transforming growth factor group.

## Abbreviations

AA	arachidonic acid
COX	cyclooxygenase
DHA	docosahexaenoic acid
EPA	eicosapentaenoic acid
GI	gastrointestinal
IFN	interferon
Ig	immunoglobulin
IL	interleukin
LCPUFA	long-chain polyunsaturated fatty acid
LOX	lipoxygenase
LT	leukotriene
PG	prostaglandin
PUFA	polyunsaturated fatty acid
SPM	specialised pro-resolving mediator
SPT	skin prick test
Th1	T-helper 1
Th2	T-helper 2
